# Plastic-Film Mulching for Enhanced Water-Use Efficiency and Economic Returns from Maize Fields in Semiarid China

**DOI:** 10.3389/fpls.2017.00512

**Published:** 2017-04-06

**Authors:** Peng Zhang, Ting Wei, Tie Cai, Shahzad Ali, Qingfang Han, Xiaolong Ren, Zhikuan Jia

**Affiliations:** ^1^The Chinese Institute of Water-Saving Agriculture, Northwest A&F UniversityYangling, China; ^2^Key Laboratory of Crop Physi-Ecology and Tillage Science in Northwestern Loess Plateau, Ministry of Agriculture, Northwest A&F UniversityYangling, China

**Keywords:** crop growth, film mulch, maize yield, rainfed area, soil temperature, soil water storage

## Abstract

Film mulch has gradually been popularized to increase water availability to crops for improving and stabilizing agricultural production in the semiarid areas of Northwest China. To find more sustainable and economic film mulch methods for alleviating drought stress in semiarid region, it is necessary to test optimum planting methods in same cultivation conditions. A field experiment was conducted during 2013 and 2014 to evaluate the effects of different plastic film mulch methods on soil water, soil temperature, water use efficiency (WUE), yield and revenue. The treatments included: (i) the control, conventional flat planting without plastic film mulch (CK); (ii) flat planting with maize rows (60 cm spacing) on plastic film mulch (70 cm wide); (iii) furrow planting of maize (60 cm spacing), separated by consecutive plastic film-mulched ridges (each 50 cm wide and 15 cm tall); (iv) furrow planting of maize (60 cm spacing), separated by alternating large and small plastic film-mulched ridges (large ridges: 70 cm wide and 15 cm tall, small ridges 50 cm wide and 10 cm tall); and (v) furrow-flat planting of maize (60 cm spacing) with a large plastic film-mulched ridge (60 cm wide and 15 cm tall) alternating with a flat without plastic film-mulched space (60 cm wide). Topsoil temperature (5–25 cm) was significantly (*p* < 0.05) higher in field plots with plastic film mulch than the control (CK), and resulted in greater soil water storage (0–200 cm) up to 40 days after planting. Maize grain yield and WUE were significantly (*p* < 0.05) higher with the furrow planting methods (consecutive film-mulched ridges and alternating film-mulched ridges) than the check in both years. Maize yield was, on average, 29% (*p* < 0.05) greater and 28% (*p* < 0.05) greater with these furrow planting methods, while the average WUE increased by 22.8% (*p* < 0.05) with consecutive film-mulched ridges and 21.1% (*p* < 0.05) with alternating film-mulched ridges. The 2-year average net income increased by 1559, 528, and 350 Chinese Yuan (CNY) ha^−1^ with the consecutive film-mulched ridges, furrow-flat planting and alternating film-mulched ridges, respectively, compared with the control (CK). We conclude that the consecutive film-mulched ridge method was the most productive and profitable for maize in this semi-arid area with limited and erratic precipitation.

## Introduction

Dryland farming, which is practiced on about one-third of the arable land the Loess plateau, Northwest China, is constrained by the semiarid growing conditions (Li and Xiao, 1992). Precipitation during the growing season occurs mainly in the form of light rain showers and rainstorm, which contribute to soil erosion and water loss through runoff. The natural rainfall regime is not effective in supplying water at critical crop growth stages and recharging soil water reserves, resulting in frequent drought (Li et al., 2001). Spring maize (*Zea mays* L.) is one of the major crops in this region, accounting for 27.3% of the total agricultural area (Liu et al., 2010), but limited water availability (Du et al., 2010; Zhou et al., 2012) and erratic precipitation often lead to low maize yields and crop failure in some cases (Gan et al., 2013). As well, low soil temperature at the seedling stage can impede maize development and growth (Ramakrishna et al., 2006). Hence, the key to stabilizing and increasing maize yields in this region is to boost WUE from precipitation. This involves better methods of capturing, reducing evaporation and alleviating low soil temperatures in spring.

Plastic film mulch is widely used as a low-cost measure to improve water retention in the soil (Wang et al., 2009), increase soil temperature (Liu et al., 2010) and reduce soil evaporation (Li and Xiao, 1992). It provides economic benefits to the farmer because it promotes crop development, achieve an early harvest and increase maize yield, according to short- and long-term research (Liu et al., 2009; Steinmetz et al., 2016). In recent years, several mulching techniques have been developed and adopted, including (1) flat planting mulched with plastic film (Wang et al., 2011), (2) alternating ridges and furrows with only the ridges mulched with plastic film (Li et al., 2001; Ren et al., 2008), and (3) alternating large and small plastic film-mulched ridges (Liu et al., 2009; Zhou et al., 2009). However, the variable hydrothermal conditions in dryland farming areas mean that different film mulch methods are not equally effective for maize production. Wang et al. (2011) did not find water accumulation from rainfall events < 10 mm when they examined flat planting with maize rows on plastic film mulch without ridges. Li et al. (2001) and Ren et al. (2008) reported greater soil water content in years with different rainfall amounts when plastic film mulch was used in a furrow-flat planting of maize (60 cm spacing) with a large plastic film-mulched ridge (60 cm wide and 15 cm tall) alternating with a flat, bare space (60 cm wide), but the grain yield did not improve. In fact, there was less grain yield in the mulched plots than the unmulched control in a rainy year (annual rainfall > 440 mm) with low temperature because the plastic film mulch trapped precipitation and resulted in high soil water storage levels in the topsoil (0–40 cm). Liu et al. (2009) and Zhou et al. (2009) argued that alternating large and small plastic film-mulched ridges had little or no effect on net income because of high costs and labor inputs, as well increased soil dryness in the deep soil profile with continuous cropping, which caused soil degradation and yield decrease.

Clearly, it is difficult to predict maize productivity in response to plastic film mulch methods, given the diverse responses in crop growth and WUE across the soil hydrological conditions present in dryland farming areas (Li et al., 2010; Gan et al., 2013), which make difficult to reach the crop productivity potential. Moreover, most previous studies have concentrated on examining the crop yield and soil water effects in farmland exposed to only one plastic film mulch practice (Ren et al., 2008; Zhou et al., 2009; Zhang et al., 2011; Gan et al., 2013). There is scant information to compare among plastic film mulch practices when cultivating the same crop under same agro-ecological conditions. With the film mulch gradually popularized in semiarid area, it is necessary to compare and then find a more sustainable and economic methods for alleviating drought stress and increasing crop yield in these regions. Therefore, in this study, we explored the effects of three different present plastic film mulch practices on spring maize production under the same cultivation conditions.

In addition, several disadvantages of plastic film mulch practices are known, i.e., placing plastic film mulch on a flat planting without ridges is ineffective to accumulate rainfall; the wide furrow covered with plastic film mulch tends to be cooler, which delays crop growth and development; it is expensive to use plastic film to mulch alternating large and small ridges, and this practices may deplete deep soil water reserves. Thus, it is necessary to test a new film mulch technique to alleviate the deficiencies of the current plastic film mulch techniques. Therefore, a new practice where consecutive plastic film-mulched ridges (each ridge measures 50 cm wide) are separated by planted furrows (each 10 cm wide without plastic film mulch) as an alternative configuration was tested in this study. The objectives of our research were: (i) to assess the effects of different plastic film mulch practices on soil water storage and temperature levels and its distribution in soil profile during the maize growing season; (ii) to explore the influence of our new plastic film mulch practice on grain yield, WUE and economic benefits in semiarid regions of the Loess plateau, China.

## Materials and Methods

### Site Description

The field experiments were conducted during 2013 and 2014 at the Dryland Agricultural Research Station, Pengyang County, Ningxia, China (106°45′N, 35°79′E and 1800 m a.s.l.). The experimental area is characterized by a semiarid, warm temperature, and continental monsoon climate. The average annual precipitation was 440 mm, and in this region ranges from 150 and 300 mm in the north to 500–700 mm in the south, more than 60% of which occurred from July to September. The annual mean temperature average was 8.1°C and the annual mean evaporation was 1100 mm, with a frost-free period of 158 days.

The field experimental was conducted on a flat field. According to the FAO/UNESCO Soil Classification (FAO/UNESCO, 1993), the soil at the experimental site was a Calcic Cambisol (sand 14%, silt 26%, and clay 60%) with relatively low fertility. Selected soil physico-chemical properties at the beginning of the experiment are presented in **Table 1**.

**Table 1:** Selected physico-chemical properties of the loess soil (0–60 cm depth) at the Dryland Agricultural Research Station, Pengyang County, China.

Soil layer	Organic matter	Available nitrogen	Available phosphorus	Available potassium	Total nitrogen	Bulk density	Porosity	Saturated moisture	pH
(cm)	(g kg^−1^)	(mg kg^−1^)	(mg kg^−1^)	(mg kg^−1^)	(g kg^−1^)	(g cm^−3^)	(%)	(%)	
0–20	8.65	63.6	12.6	161.2	1.19	1.33	49.8	37.4	8.4
20–40	7.95	44.9	7.9	117.2	0.94	1.34	49.4	36.4	8.5
40–60	7.57	46.8	6.0	102.7	1.05	1.41	46.8	38.4	8.6

*Organic matter was determined using the Walkley–Black method; The total nitrogen was determined by Kjeldahl digestion; The available phosphorus was determined using the molybdenum blue method; The available nitrogen was determined by alkaline hydrolysis method; Available potassium was determined with flame photometric method; The soil bulk density was determined using the core method, and soil porosity was calculated according to bulk density; pH was determined by potentiometry method.*

### Experimental Design and Field Management

**FIGURE 1:**
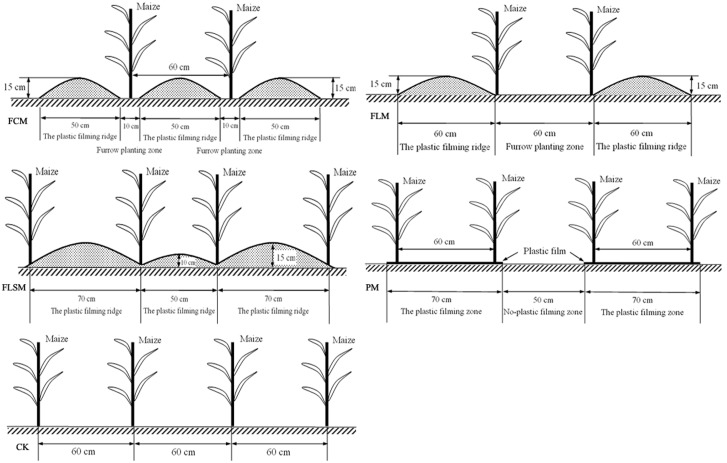
**Schematic diagram of the field layout.** CK, the control, conventional flat planting without plastic film mulch; PM, flat planting with maize rows (60 cm spacing) on plastic film mulch (70 cm wide); FCM, furrow planting of maize (60 cm spacing), separated by consecutive plastic film-mulched ridges (each 50 cm wide and 15 cm tall); FLSM, furrow planting of maize (60 cm spacing), separated by alternating large and small plastic film-mulched ridges (large ridges: 70 cm wide and 15 cm tall, small ridges 50 cm wide and 10 cm tall); FLM, furrow-flat planting of maize (60 cm spacing) with a large plastic film-mulched ridge (60 cm wide and 15 cm tall) alternating with a flat plastic film-mulched space (60 cm wide).

The experiment was a completely randomized block design with three replicates of five treatments. Plots measured 3.6 m wide and 11.0 m long and were under conventional tillage. The plots were under conventional tillage. As shown in **Figure 1**, treatments were: (i) the control, conventional flat planting without plastic film mulch (CK); (ii) the flat planting with maize rows (60 cm spacing) on plastic film mulch (PM), where the flat planting and plastic film mulch area measured 70 cm wide, with a 50 cm-wide un-mulched space between the two rows of mulched film, and maize was sown as a double row in the film; (iii) furrow planting of maize, separated by consecutive plastic film-mulched ridges (FCM), where the ridges were 50 cm wide and 15 cm high, which were covered with plastic film (70 cm wide), and the furrows were 10 cm wide for sowing a single row of maize; (iv) furrow planting of maize (60 cm spacing), separated by alternating large and small plastic film-mulched ridges (FLSM), where large ridges (70 cm in width by 15 cm in height) were alternated with small ridges (50 cm in width by 10 cm in height) and both were mulched with plastic film measuring 140 cm wide, and the two ridges were separated a furrow in which the maize was planted; and (v) furrow-flat planting of maize with a large plastic film-mulched ridge alternating with a flat without plastic film-mulched space (FLM), where the ridges covered with plastic film measured 60 cm wide and 15 cm high, and the furrows without plastic film mulch were both 60 cm wide for sowing double rows of maize in the film-side.

The plastic film was polyethylene with a thickness of 0.008 mm, which was made by the Gansu Tianbao Plastic Plant, China, and the plastic film was stability and not decomposed after crop harvested. A sketch of each plastic film mulch mode is presented in **Figure 1**.

The experimental plots were established in March 24, 2013 by plowing the field and delineating the plots. Ridges were formed in 9 of the 15 plots. Ten days before planting, basal fertilizers (150 kg N ha^−1^ and 150 kg P_2_O_5_ ha^−1^) were applied across the unridged plots (six plots: CK and PM treatments) and incorporated manually with a spade to 5 cm depth, or spread in the furrow (nine plots: FLSM, FLM and FCM treatments) and mixed manually to a depth of 5 cm with a spade. Plastic film mulch was placed on the soil surface according to the configurations in **Figure 1** within 2 days after fertilization.

Maize (Dafeng 30) was sown at a rate of 75 000 plants ha^−1^ on April 14, 2013 using a hole sowing (3 cm in diameter) machine. In addition, 150 kg N ha^−1^ was applied as a top dressing in late June after maize planting. Crops were harvested from the plots on September 28, 2013. After harvesting the maize, the configuration and mulch were retained in the same location on each of the plots, but the maize stalks were removed and the plastic film was cleared up to 30 days before subsequent sowing operation (March 27, 2014), corn planting in April 28, 2014, the post-emergence fertilization on June 29, and harvest on October 4, 2014, while the process and method was similar as that in 2013. Artificial irrigation was not provided throughout the years of the experiment and weeds were controlled manually during each crop growth season, as required.

### Sampling and Measurement

During the experimental period, rainfall data were recorded using an automatic standard weather station (WS-STD1, Delta-T, UK) located at the experimental site.

Mercury-in-glass geothermometers (Hongxing Thermal Instruments, China) were placed between the two maize plants in each treatment plots at soil depths of 5, 10, 15, 20, and 25 cm to determine the soil temperature. Soil temperatures were recorded at 08:00, 10:00, 12:00, 14:00, 16:00, 18:00, and 20:00 h each day at 10, 40, 70, 100, 130, and 170 days after planting. Mean daily soil temperature was calculated as the average of readings of 3 days.

Soil water content was determined at 20 cm increments, to a depths of 200 cm at 10, 40, 70, 100, 130, and 170 days after planting. A 54 mm diameter steel core-sampling tube was inserted manually between two plants, located in the middle rows of each plot, at three locations per plot. Soil cores were weighed wet, dried in a fan-assisted oven at 105°C for 48 h, and the dry weighed assessed to determine the soil water content (Ferraro and Ghersa, 2007). The gravimetric water content was multiplied by soil bulk density to obtain the volumetric water content.

The soil water storage was calculated using Eq (1) as follows:

(1) $$\begin{array}{c} S_{w}=h\times d\times b\%\times 10 \end{array}$$

where S_w_ (mm) is the averaged values of soil moisture; h (cm) is soil layer depth; *d* (g cm^−3^) is soil bulk density in different soil layer, and b% is the percentage of soil moisture in weight.

In 2013 and 2014, 30 representative maize plants per plot were used for each measurement at harvest, whilst the ear length, ear diameter, seed number per ear, and 100-kernel weight was recorded.

The WUE was estimated as the grain yield divided by the growing season evapotranspiration (ET, mm) (Hussain and Al-Jaloud, 1995), as follows:

(2) $$\begin{array}{c} WUE=Yield/ET \end{array}$$

where *ET* was calculated as (Li et al., 2013):

(3) $$\begin{array}{c} ET=W_{1}-W_{2}+P \end{array}$$

where W_1_ (mm) is the soil water storage for the 0–200 cm soil depth before sowing, W_2_ (mm) is the soil water storage for the 0–200 cm soil depth at harvesting, and *p* (mm) is the rainfall during the maize growing season.

The harvest index (HI) based on maize grain yield and biomass yield was calculated as follows:

(4) $$\begin{array}{c} HI=\frac{Y_{g}}{Y_{b}} \end{array}$$

where Y _g_ (kg ha^−1^) is the grain yield, and Y _b_ (kg ha^−1^) is the biomass yield.

Net economic profit for each treatment was calculated using the following equations:

(5) $$\begin{array}{c} OV=Y_{g}\times P_{g}+Y_{b}\times P_{b} \end{array}$$

(6) $$\begin{array}{c} IV=LC\times MC+MCC\times SFC \end{array}$$

(7) $$\begin{array}{c} O/I=\frac{OV}{IV} \end{array}$$

(8) $$\begin{array}{c} NI=OV-IV \end{array}$$

where *OV* is the output value (Chinese Yuan ha^−1^), Y _g_ (kg ha^−1^) is the grain yield, Y _b_ (kg ha^−1^) is the biomass yield, P_g_ and P_b_ is the local price of maize grain and biomass (Chinese Yuan ha^−1^), *IV* is the total input value (Chinese Yuan ha^−1^), *LC* is the labor cost (Chinese Yuan ha^−1^), *MC* is the film mulching cost (Chinese Yuan ha^−1^), *MCC* is the machine-cultivation cost (Chinese Yuan ha^−1^), *SFC* is the seed and fertilizer cost (Chinese Yuan ha^−1^), and *NI* is the net income (Chinese Yuan ha^−1^).

### Statistical Analysis

Data values were analyzed by residual test method before statistical analysis, and the data met the assumption of homogeneity of variances and followed the normal distribution. Significant differences were determined by ANOVA, and multiple comparison analysis were performed with Tukey HSD test (*p* < 0.05). All the analyses were performed with a confidence level of 95% by using SPSS 13.0 (SPSS Inc. Chicago, IL, USA). All figures were prepared using Sigma Plot 12.5.

## Results

### Rainfall

**FIGURE 2:**
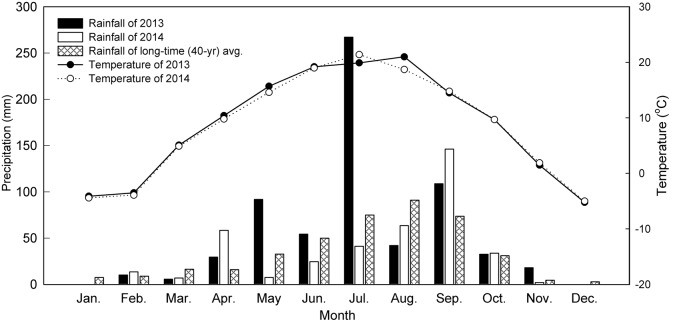
**Distribution of monthly precipitation and air temperature during 2013–2014 at the Dryland Agricultural Research Station, Pengyang County, China**.

Precipitation during the maize growing seasons was 594 in 2013 and 342 mm in 2014, while the 20-year average was 339 mm (**Figure 2**), indicating that 2013 was a wetter-than-normal season while 2014 was a normal season. It also showed that the rainfall was erratic and has different patterns each year, but temperature pattern was relatively consistent during the two growing season (**Figure 2**).

### Soil Temperature

**FIGURE 3:**
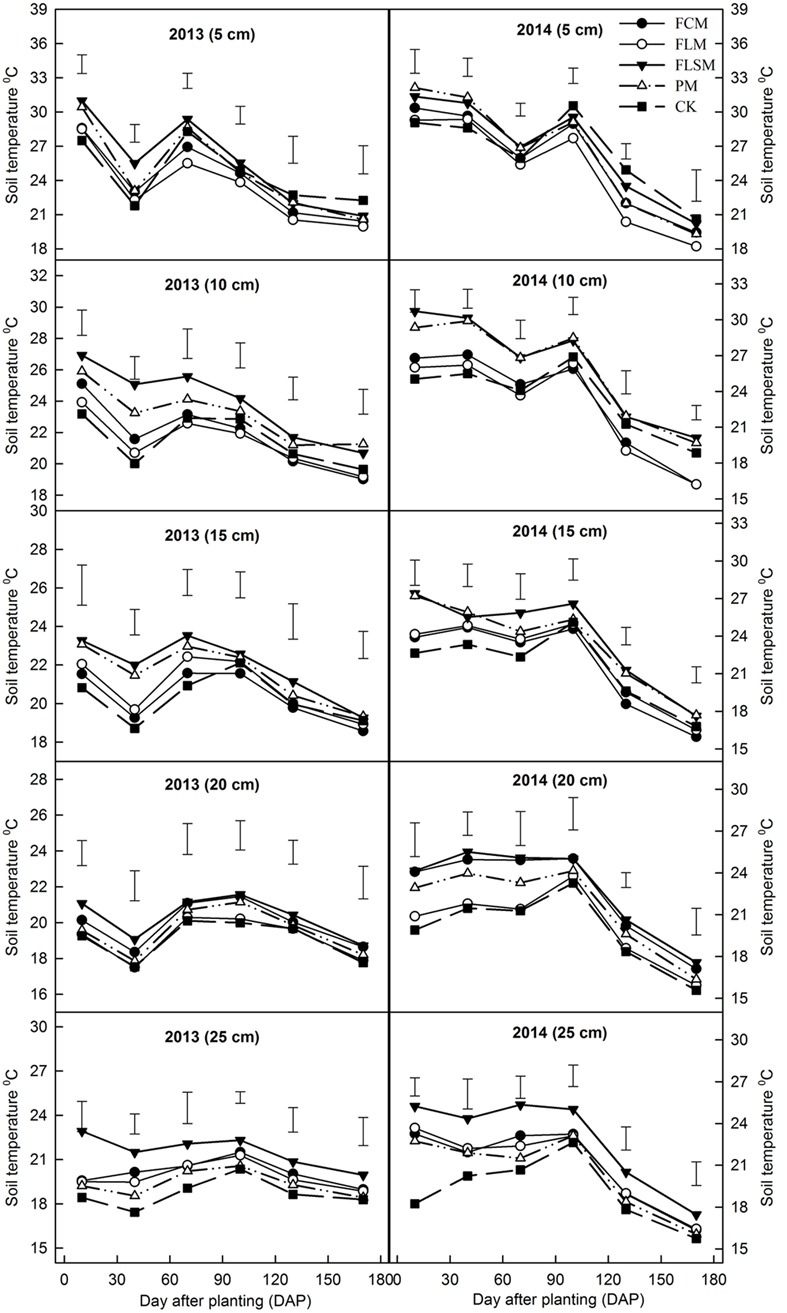
**Effects of different film mulching treatments on soil temperature at different soil depths and times in 2013–2014 at the Dryland Agricultural Research Station, Pengyang County, China.** CK, the control, conventional flat planting without plastic film mulch; PM, flat planting with maize rows (60 cm spacing) on plastic film mulch (70 cm wide); FCM, furrow planting of maize (60 cm spacing), separated by consecutive plastic film-mulched ridges (each 50 cm wide and 15 cm tall); FLSM, furrow planting of maize (60 cm spacing), separated by alternating large and small plastic film-mulched ridges (large ridges: 70 cm wide and 15 cm tall, small ridges 50 cm wide and 10 cm tall); FLM, furrow-flat planting of maize (60 cm spacing) with a large plastic film-mulched ridge (60 cm wide and 15 cm tall) alternating with a flat plastic film-mulched space (60 cm wide). Error bars indicate l.s.d. value.

Soil temperature at 5 cm depth was significantly greater in plots with plastic film mulch than the control plots during early maize growth (up to 40 DAP) by as much as 1.9°C in 2013 and 1.7°C in 2014 (**Figure 3**). As the maize canopy developed during the growing season, the soil temperature was cooler in the plastic film mulch plots, and the mean soil temperature of at 5 cm depth of plastic film mulch plots was lower than CK (after to 130 DAP) by 1.5°C in 2013 and 2.1°C in 2014. At all growth stages, soil temperature at 10 and 15 cm depth was warmer in the FLSM treatment than the CK (up to 2.1°C in 2013 and 2.6°C in 2014) and in the PM treatment than the CK (by as much as 1.5°C in 2013 and 2.2°C in 2014). Soil temperature at 10 and 15 cm depths was warmer in the FCM and FLM treatments than the CK from 0 to 70 DAP, and there after the temperature was similar in these treatments. During the growing season, soil temperature at 20 cm depth was greater in plastic film mulch treatments than the CK plots, by as much as 0.6°C in 2013 and 1.6°C in 2014, and we also recorded higher soil temperature at 25 cm depth with plastic film mulch than the CK, up to 1.2°C warmer in 2013 and 1.8°C hotter in 2014.

### Soil Water Storage

**FIGURE 4:**
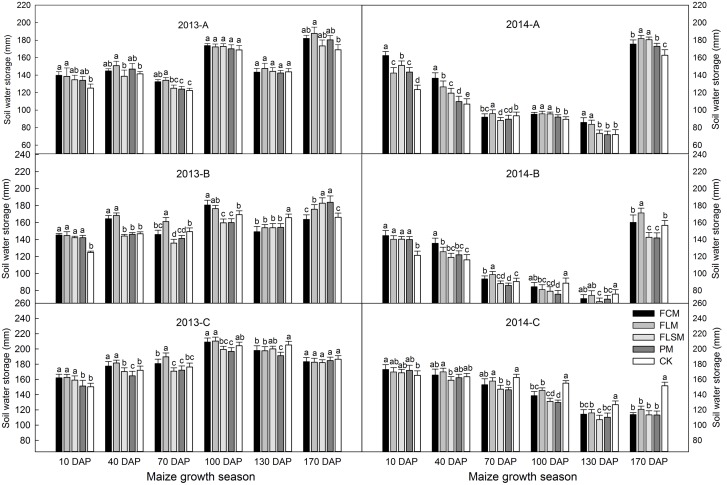
**The soil water storage dynamics in 0–60, 60–120, and 120–200 cm layers with different film mulching treatments during maize growing season in 2013–2014 at the Dryland Agricultural Research Station, Pengyang County, China.** (A–C) Soil water storage at 0–60 (A), 60–120 (B), and 120–200 (C) cm layers, respectively. CK, the control, conventional flat planting without plastic film mulch; PM, flat planting with maize rows (60 cm spacing) on plastic film mulch (70 cm wide); FCM, furrow planting of maize (60 cm spacing), separated by consecutive plastic film-mulched ridges (each 50 cm wide and 15 cm tall); FLSM: furrow planting of maize (60 cm spacing), separated by alternating large and small plastic film-mulched ridges (large ridges: 70 cm wide and 15 cm tall, small ridges 50 cm wide and 10 cm tall); FLM, furrow-flat planting of maize (60 cm spacing) with a large plastic film-mulched ridge (60 cm wide and 15 cm tall) alternating with a flat plastic film-mulched space (60 cm wide). Data are means ± SD (*n* = 3). Bars with different lower case letters indicate significant differences among treatments for each year (Tukey HSD test, *p* < 0.05).

Plastic film mulch improved soil water storage during the early maize growth. From 0 to 10 DAP in 2013, based on statistical analysis (Turkey test), the soil water storage in the 0–60 cm depth was greater in plots with plastic film mulch than the CK plots by as much as 11.7% (FCM treatment, *p* < 0.05), 10.7% (FLM treatment, *p* < 0.05), 7.5% (FLSM treatment), and 7.2% (PM treatment), respectively (**Figure 4**). Soil water reserves were replenished by 121.4 mm rainfall during April to May, and depleted by maize water use, which resulted in no significant difference among the treatments at 40 DAP. Only FCM and FLM had significantly (*p* < 0.05) more soil water storage than the CK (8.2 and 9.5%, respectively) at 70 DAP. From 100 to 130 DAP, several rainfall events delivered more than 260 mm of precipitation (45% of annual rainfall) which replenished the soil water reserves, and resulted in 7.1% more soil water storage, on average, in the plastic film mulch treatments than the CK by 170 DAP.

From 0 to 10 DAP in 2013, based on statistical analysis (Turkey test), the soil water storage in the > 60–120 cm depth was significantly (*p* < 0.05) greater in plots with plastic film mulch than the CK plots by 15.0%. Soil water reserves were depleted by maize water use, which resulted in only FLM and FCM had significantly (*p* < 0.05) more soil water storage than the CK (12.1 and 14.7%, respectively) at 40 DAP. Only FLM had significantly (*p* < 0.05) more soil water storage than the CK by as much as 7.9% at 70 DAP, and 4.2% at 100 DAP. At 130 DAP, the soil water storage in the >60–120 cm depth with plastic film mulch than the CK plots by 7.8%. Soil water reserves were replenished by rainfall increase and consumption decrease, and resulted in 6.2% more soil water storage, on average, in the plastic film mulch treatments than the CK at 170 DAP.

The trend of soil water storage of each plots at >120–200 cm depth increased at 0–100 DAP and then decreased (**Figure 4**). From 0 to 100 DAP in 2013, only FLM and FCM had more soil water storage than the CK by as much as 3.8 and 5.9% (*p* < 0.05), respectively. At 130 DAP, all plastic film mulch plots was lower than CK by 4.1%, and no significant difference among the treatments at 170 DAP.

Less rainfall during the maize growth stage, which resulted in the soil water storage in 2014 was lower than that in 2013 at each soil depth (**Figure 4**). Plastic film mulch improved soil water storage during the early maize growth. Based on the analysis of statistical results, in 2014, the soil water storage in the 0–60 cm depth was greater in plots with plastic film mulch than the CK plots by as much as 21.4% (*p* < 0.05) at 10 DAP, 15.1% (*p* < 0.05) at 40 DAP, and 5.9% at 100 DAP. Soil water reserves were replenished by 63.6 mm rainfall during 100–130 DAP, which resulted in only FLM and FCM had significantly (*p* < 0.05) more soil water storage than the CK (19.2 and 16.2%, respectively). At 170 DAP, 146.3 mm rainfall of precipitation was replenished the soil water reserves, and resulted in 9.2% more soil water storage, on average, in the plastic film mulch treatments than the CK.

From 0 to 40 DAP, the soil water storage in the >60–120 cm depth was significantly (*p* < 0.05) greater in plots with plastic film mulch than the CK plots by 12.4%. Soil water reserves were depleted by maize water use, which resulted in all plastic film mulch plots was lower than CK by 9.6% at 100 DAP. At 130 DAP, all plots soil water storage was lowest (range 100–120 mm), and no significant difference among the treatments. Soil water reserves were replenished by rainfall, and resulted in only FLM and FCM had more soil water storage than the CK (2.3 and 9.3%, respectively) at 170 DAP.

Plastic film mulch decreased soil water storage during the late maize growth. From 70 to 170 DAP in 2014, the soil water storage in the >120–200 cm depth was lower in plots with plastic film mulch plots w than the CK by 18.3%, no significant difference among the treatments.

### Crop Development

Early maize growth was accelerated with plastic film mulch treatments compared to the CK in both years (**Table 2**). This resulted in a shorter time to physiological maturity, from 10–16 days in 2013 and 5–12 days in 2014. Plastic film mulch treatments advanced the sowing-emergence time by 3–8 days in 2013 and 4–8 days in 2014. Similarly, the emergence-jointing stage was 4–8 days earlier in 2013 and 3–7 days earlier in 2014. The jointing-tasselling stage was also advanced, by 6–9 days in 2013 and 3–9 days in 2014. As a result, the milk-maturity stage was 5–12 days longer in 2013 and 7–14 days longer in 2014.

**Table 2:** Maize crop development (day) under different mulch plots during 2013–2014 at the Dryland Agricultural Research Station, Pengyang County, China.

Year	Treatments	Sowing-Emergence	Emergence- Jointing	Jointing-Trumpeting	Trumpeting- Tasseling	Tasseling- Blooming	Blooming-Milking	Milking-Maturity	Total
2013	FCM	7	28	21	21	4	7	65	153
	FLM	10	30	20	22	4	10	59	155
	FLSM	5	26	20	20	4	8	66	149
	PM	5	28	18	21	4	11	65	152
	CK	13	34	23	25	5	11	54	165
2014	FCM	8	37	17	19	3	6	56	146
	FLM	10	41	18	21	4	8	51	153
	FLSM	6	39	16	17	3	7	58	146
	PM	8	39	17	17	3	6	56	146
	CK	14	44	20	22	4	10	44	158

*CK, the control, conventional flat planting without plastic film mulch; PM, flat planting with maize rows (60 cm spacing) on plastic film mulch (70 cm wide); FCM, furrow planting of maize (60 cm spacing), separated by consecutive plastic film-mulched ridges (each 50 cm wide and 15 cm tall); FLSM, furrow planting of maize (60 cm spacing), separated by alternating large and small plastic film-mulched ridges (large ridges: 70 cm wide and 15 cm tall, small ridges 50 cm wide and 10 cm tall); FLM, furrow-flat planting of maize (60 cm spacing) with a large plastic film-mulched ridge (60 cm wide and 15 cm tall) alternating with a flat plastic film-mulched space (60 cm wide).*

### Agronomic Properties

Maize grown in plastic film mulch treatments had bigger ears, which were from 5.8 to 9.0% longer and had a 8.1–12.9% lager diameter than those from the CK plots, based on maize samples collected during the 2013 and 2014 growing seasons (**Table 3**). Based on statistical analysis (Turkey test), grain weight increased significantly (*p* < 0.05) when maize was grown in plastic film mulch, and the 100-kernel weight was 23% greater with FCM, 16% higher with FLM, 24% more in FLSM, and 5.9% greater with PM than the CK plots (**Table 3**). Similarly, the grain number per ear and shelling percentage of maize were improved significantly (*p* < 0.05) when maize was grown on plastic film mulch (on average, 5.4% more grains per ear and 4.5% higher shelling percentage) than in the CK plots.

**Table 3:** Effects of different mulch plots on agronomic properties of maize during 2013–2014 at the Dryland Agricultural Research Station, Pengyang County, China.

Year	Treatments	Ear length (cm)	Ear diameter (cm)	100-kernel weight (g)	Grain number per ear	Shelling (%)
2013	FCM	20.19a	51.42ab	42.56a	608.48a	86.30a
	FLM	18.73b	49.04b	39.73ab	585.79bc	82.58b
	FLSM	19.26ab	51.82a	42.15a	595.96abc	84.10ab
	PM	19.04b	50.23ab	35.19c	605.31ab	86.11a
	CK	18.28b	49.54ab	36.03b	582.71c	81.91b
2014	FCM	19.52a	52.38ab	33.73a	640.13a	80.38a
	FLM	20.41a	51.68ab	32.50ab	631.33a	81.31ab
	FLSM	20.10a	52.75a	34.85a	633.09a	82.82a
	PM	19.49a	50.40b	30.55b	578.39b	80.40b
	CK	18.15a	46.34c	26.07c	574.35b	77.01c

*CK, the control, conventional flat planting without plastic film mulch; PM, flat planting with maize rows (60 cm spacing) on plastic film mulch (70 cm wide); FCM, furrow planting of maize (60 cm spacing), separated by consecutive plastic film-mulched ridges (each 50 cm wide and 15 cm tall); FLSM, furrow planting of maize (60 cm spacing), separated by alternating large and small plastic film-mulched ridges (large ridges: 70 cm wide and 15 cm tall, small ridges 50 cm wide and 10 cm tall); FLM, furrow-flat planting of maize (60 cm spacing) with a large plastic film-mulched ridge (60 cm wide and 15 cm tall) alternating with a flat plastic film-mulched space (60 cm wide). Values followed by the different lowercase letter in the same row indicate significant differences among treatments for each year (Tukey HSD test, *p* < 0.05).*

### Yield and Water Use Efficiency

Grain and biomass yield of maize was significantly influenced by the different plastic film mulch plots over the 2 years, with the higher yield recorded in 2013 and the lower in 2014 (**Table 4**). In 2013, the maize grain yields for each of the treatments were ranked as follows: FCM > FLSM > PM > CK > FLM, and the results of the statistical analysis (Turkey test) showed that the maize yield with FCM, FLSM, and PM treatments were significantly (*p* < 0.05) higher than CK by 20.3, 18.0, and 11.2%, respectively. While in 2014, the yields were ranked as follows: FLSM > FCM > FLM > PM > CK, the mean maize yields with plastic film mulch plots were significantly (*p* < 0.05) higher than CK by 34.9%.

**Table 4:** Effects of different mulch plots on the grain yield, biomass yield, harvest index (HI), evapotranspiration (ET), and water use efficiency (WUE) of maize in 2013–2014 at the Dryland Agricultural Research Station, Pengyang County, China.

Year	Treatments	Grain yield (kg ha^−1^)	Biomass yield (kg ha^−1^)	HI	ET (mm)	WUE (kg ha^−1^ mm^−1^)
2013	FCM	16200a	24422ab	0.67a	522.02a	31.09a
	FLM	12957c	22769b	0.57b	509.66a	25.42b
	FLSM	15896a	25271a	0.63ab	505.38a	31.11a
	PM	14976b	25031ab	0.60ab	450.17b	33.27a
	CK	13470c	23432ab	0.58b	490.91a	27.44b
2014	FCM	13509a	24359ab	0.46d	475.86bc	28.39a
	FLM	13421a	23761ab	0.57b	478.90ab	28.12a
	FLSM	13671a	26242a	0.53bc	496.12ab	27.55a
	PM	11169b	20986bc	0.64a	500.59a	22.32b
	CK	9594b	19917c	0.48cd	457.06c	21.01b

*CK, the control, conventional flat planting without plastic film mulch; PM, flat planting with maize rows (60 cm spacing) on plastic film mulch (70 cm wide); FCM, furrow planting of maize (60 cm spacing), separated by consecutive plastic film-mulched ridges (each 50 cm wide and 15 cm tall); FLSM, furrow planting of maize (60 cm spacing), separated by alternating large and small plastic film-mulched ridges (large ridges: 70 cm wide and 15 cm tall, small ridges 50 cm wide and 10 cm tall); FLM, furrow-flat planting of maize (60 cm spacing) with a large plastic film-mulched ridge (60 cm wide and 15 cm tall) alternating with a flat plastic film-mulched space (60 cm wide). Values followed by the different lowercase letter in the same row indicate significant differences among treatments for each year (Tukey HSD test, *p* < 0.05).*

The biomass yields have the similar trends with the grain yield. Based on statistical analysis (Turkey test), the mean biomass yield with plastic film mulch (except FLM) was significant higher than CK by 6.3% in 2013 and 19.7% (*p* < 0.05) in 2014.

The HI was ranging from 0.57 and 0.67 in 2013, only FCM significantly higher than CK (15.5%). While in 2014, the results of the statistical analysis (Turkey test) showed that the plastic film mulch plots (except FCM) were all significantly (*p* < 0.05) higher than CK, i.e. the FLM, FLSM, and P treatments significantly increased by 18.8, 10.4, and 33.3%, respectively.

The WUE have the similar trends with the yield. Based on statistical analysis (Turkey test), the WUE of plastic film mulch plots (except FLM) were all significantly higher than CK in 2013, i.e., significantly (*p* < 0.05) increased by 13.3, 13.4, and 21.2% with FCM, FLSM, and PM, respectively. In 2014, the WUE was greater in plots with plastic film mulch than the CK plots by 26.6%.

Because of the difference of rainfall, the ET of each treatment was higher in 2013 than in 2014. In 2013, only FCM significant (*p* < 0.05) increased by 6.3% compared with CK. While in 2014, the plastic film mulch plots were all higher than CK, average increased by 6.7%.

### Economic Benefit

There were obvious differences in the input costs of the various plastic mulch plots, because of the use of mulching amounts and labor (**Table 5**). The 2-year average input cost was ranked as follows: FLSM > FCM > PM > FLM > CK, while the output value followed by FLSM > FCM > FLM > PM > CK. The output/input ratio of each plastic film mulch plots was lower than CK (average decreased by 15.2%). Net income of FCM, FLM, FLSM treatments were higher than that of CK, i.e., increased by 1559, 528, and 350 CNY ha^−1^, respectively. While PM treatment was lower than CK by 538 CNY ha^−1^.

**Table 5:** Average economic output and input costs for maize production during 2013–2014 at the Dryland Agricultural Research Station, Pengyang County, China.

Treatments	LC	MC	MCC	SFC	IV	OV	O/I	NI	NID
FCM	2700	1800	1500	3135	9135	20264	2.22	11129	1558.6
FLM	2700	720	1500	3135	8055	18153	2.25	10098	527.5
FLSM	3600	2160	1500	3135	10395	20316	1.95	9921	350
PM	3600	720	1500	3135	8955	17988	2.01	9033	-538
CK	1800	0	1500	3135	6435	16006	2.49	9571	0

*CK, the control, conventional flat planting without plastic film mulch; PM, flat planting with maize rows (60 cm spacing) on plastic film mulch (70 cm wide); FCM, furrow planting of maize (60 cm spacing), separated by consecutive plastic film-mulched ridges (each 50 cm wide and 15 cm tall); FLSM, furrow planting of maize (60 cm spacing), separated by alternating large and small plastic film-mulched ridges (large ridges: 70 cm wide and 15 cm tall, small ridges 50 cm wide and 10 cm tall); FLM: furrow-flat planting of maize (60 cm spacing) with a large plastic film-mulched ridge (60 cm wide and 15 cm tall) alternating with a flat plastic film-mulched space (60 cm wide). LC, labor costs [Chinese yuan (CNY) ha^−1^]; MC, film mulching costs (CNY ha^−1^); MCC, machine-cultivation costs (CNY ha^−1^); SFC, seed and fertilizer costs (CNY ha^−1^); IV, input value (CNY ha^−1^); OV, output value (CNY ha^−1^); O/I, output/input; NI, net income (CNY ha^−1^); NID, net income difference (CNY ha^−1^) compared with CK. Labor cost = 80 CNY per person per day; plastic film cost = 12 CNY kg^−1^; maize seed price = 1.20 CNY kg^−1^; maize straw price = 0.1 CNY kg^−1^.*

## Discussion

Field management practices affect the soil surface conditions as well as influencing the soil water and thermal status, which play important roles in crop growth and development during dryland farming (Chakraborty et al., 2008). In the loess plateau region of northwest China, intensive cultivation systems are employed but with poor soil management strategies (Wang et al., 2009; Zhang et al., 2009). The results of the present study demonstrate that plastic film mulch had positive effects on the soil water storage, soil temperature, and crop yield. Therefore, appropriate plastic film mulch managements are very important for sustainable agricultural development in these semiarid areas, and also applied to other similar regions of the world.

### Soil Temperature

A suitable soil temperature is a basic requirement for crops to maintain the root activity, while changes in the root morphology may affect crop yield (Stone et al., 1999). Several studies have shown that suitable temperatures during the early stage of crop growth can greatly accelerate grain germination and crop yield (Ren et al., 2016). Our results showed that irrespective of depth, the effects of different plastic film mulch plots on the soil temperature were greater during the early growth stages, with a mean increase of 0.8°C, which was probably because the plant canopy was sufficiently small and sparse during the early stage of crop development so the majority of the plastic film area received solar energy to warm the topsoil (Liu et al., 2010; Gan et al., 2013). It was shown that the plastic film mulch could provide a favorable soil temperature for crop emergence. By contrast, after the full establishment of the plant canopy during the middle and later growth stages, the soil temperature increased little under plastic film mulch compared with the uncovered plots (CK), and because of the higher soil water storage caused low soil temperature, the FLM and FCM plots lower than CK in the 5–15 cm soil depth at 70 DAP, because the FLM and FCM treatments accumulated rainwater by ridge and furrow systems. We also showed that the effects of FLM and FCM on soil temperature increasing was significant in deeper (20–25 cm depth) layers compared with the surface layers of the soil (**Figure 3**), which agreed with the findings of Ren et al. (2008) and Li et al. (2013). This support a favorable soil microclimate for maize root proliferations to increasing soil WUE (Osuji, 1990). By contrast, the soil temperature was consistently higher in each soil layer with FLSM and PM, because the film covered the entire maize planting area, thereby preventing water exchange between the soil and air to reduce the latent heat flux (Liu et al., 2009, 2010), which led to the crop grew quickly and consumed lots of soil water to destroyed soil water balance.

### Soil Water Storage

Many studies have indicated that plastic film mulch could significantly reduce soil water evaporation and water erosion, thereby increasing the precipitation use efficiency in rain-fed farming systems (Ramakrishna et al., 2006; Turner et al., 2011; Gan et al., 2013). If the soil is dry during the seedling stage, the seeds cannot absorb sufficient water and germination may be impossible or delayed, while root productions might be decreased after seed germination, thereby affecting aboveground growth and seed yield (Ren et al., 2016). Our results showed that the plastic film mulch plots significantly increased the soil water storage (0–200 cm) compared with CK during the early growth stage (0–40 DAP), especially in FLM and FCM plots, and similar results were also reported by Li et al. (2013). This is mostly because the plant canopy is small in the early stage, and there is high water evaporation from the bare soil, but plastic film can significantly prevent the soil water exchange between the soil and air to decrease the evaporation of soil water, which provided more favorable condition for seedling growth by spring crops in the test area where the average annual temperature was only 8.1°C (Ramakrishna et al., 2006). In our research, during elongation in the maize growth stage, the soil water storage of FLSM/PM plots was lower than FLM/FCM at 0–60 cm depth, especially in the year with less rainfall (2014). There are two possible explanations for this difference: plastic film mulch on all maize grow area produced a higher soil temperature so the plants grew quickly and consumed more soil water; or most of the rainfall occurred as light rains (<10 mm) during this stage and 85% of the precipitation was lost as runoff (Ren et al., 2009), the ridge and furrow system (FLM and FCM treatments) using mulching ridges to accumulated rainwater, which increased the penetration of light rain into deep soil (Li and Gong, 2002; Tian et al., 2003). Moreover, we showed that the soil water storage in the deeper soil layer (>60–200 cm) was lower under the plastic film mulch plots than CK when the maize entered the reproductive stage, probably because most of water consumed in these stages for maize growth come from the deeper soil layer (Gan et al., 2013), and the abundant rainfall during the reproductive stage (more than 70% of the growing season rainfall occurs from July to September) could not infiltrate to the deeper layers rapidly, which also led to water deficit in the deeper layers under plastic film mulch plots.

Ren et al. (2008) observed that the most obvious effects of the ridge and furrow system on the soil water storage level occurred with annual precipitation between 230 and 440 mm, whereas there were no significant effects when the rainfall exceeded 440 mm. Li et al. (2001) also found that the ridge and furrow system decreased the soil water storage level as the precipitation increased, which agreed with our results. We found that the FLM and FCM treatments significantly increased the soil water storage in the 0–120 cm layer compared with FLSM and PM in 2014, which agreed with the results reported by Ren et al. (2010), who showed that the ridge and furrow system concentrates the rainfall and forced deeper penetration in the soil to reduce evaporation but also lateral moved into the ridges to retain soil water. In addition, it is possible that FLSM and PM consumed more soil water than FLM and FCM caused by quickly crop development.

### Crop Development

Previous studies have demonstrated that plastic film mulch can increase the soil temperature (Li et al., 2013) and soil water content (Li et al., 2001), thereby reducing germination time and promoting crop growth and development to increase grain yield. Similarly, we found that the plastic film mulch plots clearly increased soil temperature in early stage, and advanced the emergence and maturity stages compared with CK. The emergence stage under FLM/FCM plots were delayed compared with FLSM/PM by 2–5 days, because the better soil water conditions with FLM and FCM treatments lead to low temperature (Li et al., 2001). In addition, it was also associated with the maize seeding location, planting on plastic film or film-side. The milking-maturity stage was extended with the plastic film mulch plots (especially with FCM, FLSM, and PM), which agreed with the results reported by Liu et al. (2010), who observed that plastic film mulch plots promoted crop transpiration with little soil evaporation to allow the accumulation of a greater biomass during the early growth stages, while the development of plants accelerated from seedling emergence to physiological maturity, and the milking stage extended to increase the maize yield.

### Water Use Efficiency (WUE)

It has been widely reported that plastic film mulch can significantly increase the WUE (Raeini-Sarjaz and Barthakur, 1997; Li et al., 2010). Similarly, in our study, the plastic film mulch plots significantly increased the WUE by 10.5–22.8%. Surface plastic film mulch enhances the soil moisture regime by controlling evaporation from the soil surface (Raeini-Sarjaz and Barthakur, 1997; Wang et al., 2009), which improve infiltration and soil water retention, as well as providing a favorable soil microclimate for seedling emergence (Liu et al., 2010), and root proliferation (Osuji, 1990). Under each treatment (expect FLM), the WUE was higher in 2013 than 2014 because more abundant rainfall led to significantly higher maize yields in 2013 compared with 2014 (**Table 4**). In addition, nearly 50% of the rainfall occurred during late-September to the beginning-October in 2014, so is could not be used by the maize crop and it only increased the ET, thereby decreasing the WUE. Our results also demonstrated that the effect of plastic film mulch on the WUE was higher in normal season than a wetter-than-normal season, especially in FLM and FCM plots, which agreed with Li et al. (2001) and Ren et al. (2008). The optimum rainfall amount for ridge and furrow harvesting systems is 230–440 mm and there are no significant improvements in the WUE when the rainfall exceeds 440 mm.

### Grain Yield

The better grain yield response of plastic film mulch was largely due to improved topsoil temperature and soil moisture conditions through better utilization of low intensity rainfall (Cook et al., 2006; Li et al., 2008; Ren et al., 2008). Meteorological variations meant that there were differences in grain yield of maize during 2 years. Gan et al. (2013) reported that the yield improvement obtained with plastic film mulch was better when less rainfall occurred during the growing season. In our research, the grain yield with plastic film mulch were 11.4 and 34.9% higher than CK in 2013 and 2014, respectively. Thus, in a wetter-than-normal year, the soil temperature becomes the key factor that constrains crop growth instead of soil water, and maintain an optimum temperature could produce higher crop yield (Chakraborty et al., 2008). A previous study showed that different planting patterns can affect crop agronomic properties, thereby leading to changes of crop yield in the field (Zhang et al., 2007). The results found that the main increase in the maize yield under plastic film mulch plots was attributable to the higher 100-kernel weight, where this effect was particularly pronounced during the drought or average-rainfall year, i.e., 2014. The rate at which the mulched soil dried was slow and water was conserved at lower depths, and thus the availability of water was maintained for a relatively longer time during the productive growth period, particularly in the milking stage (Zhang et al., 2007; Li et al., 2008). Furthermore, plastic film mulch plots increased the heat available to maize, which is crucial for crop production in semiarid regions (Liu et al., 2010). The yield enhancements differed according to the mulching and configuration of different practice. The FCM plots (furrow planting of maize, separated by consecutive 50 cm wide and 15 cm tall plastic film-mulched ridges) had the best yield increasing effect, an average increased by 3323 kg ha^−1^ (28.8%) over 2 years. The following better treatment for yield enhancement was obtained for FLSM, and then FLM and PM. This was mainly because when the furrow width was too wide, the soil temperature increased relatively low, and not formed ridges were relatively low amounts of precipitation collected, thereby affecting the maize yield increase. Therefore, the optimum plastic film mulch planting would be FCM (furrow planting of maize, separated by consecutive 50 cm wide and 15 cm tall plastic film-mulched ridges) in the semi-arid dryland agricultural regions, which were characterized by a semiarid (annual rainfall ranges from 300 to 500 mm), warm temperature (annual temperature ranges from 5 to 10°C), and continental monsoon climate.

### Economic Benefit

Besides the improvement of yield increasing effect, the economic benefit effect of planting practice is another factor need to be considered. Economic benefit is one of the most effective evaluation indices for crop management practices, which is the most concerned by farmers. The cost of film plots would be higher than CK by about 2700 Chinese Yuan ha^−1^ (including the costs of labor and the plastic film) every year. The costs of film were higher in FLSM and FCM, because the higher film areas. Notably the labor costs were significantly higher in FLSM and PM, because the FLSM and PM were sowing under the film, and need to releasing seedlings manually, which leads to lower out/put (O/I). However, farmers often give little consideration to the labor cost, which including sowing seed, fertilizing, forming the ridges, mulching, and other field management costs in agricultural production in our experiment area. The film mulch could decrease the infection of diseases and pests in farmland, which also helpful to reduce input value. Plastic film is a relatively low-cost material and many types of plastic film can be readily found everywhere in the world which will be recycled. In this research, the most important output value from the plots was the maize grain, the market of which price stability between and within seasons in northwest China. The corresponding output value (OV) of plastic film mulch plots would increase about 1982-4310 Chinese Yuan ha^−1^ (maize price is about 1.2 Chinese Yuan kg^−1^) in average years, especially FCM and FLSM plots. Although the FCM plots needs some investment, it can be offset by growing cash crops, and get a high net income (NI) and highest net income difference (NID), and it is an option with high potential to increase crop sustainability in dryland farming system. This indicated that the FCM methods (furrow planting of maize, separated by consecutive 50 cm wide and 15 cm tall plastic film-mulched ridges) has a great potential to be widely adopted by farmers in the future under semi-arid climate, and it could serve as a new model for spring maize production for small holder farmers in semi-arid regions. However, while producing huge benefits, plastic film mulch technology has also brought on a series of environment pollution hazards. Therefore, we can combine biodegradable film to use FCM method in the future to control residual mulch pollution.

## Conclusion

The benefits of maize with plastic film mulch in semiarid agricultural systems are enormous, though the effects between plastic film mulch plots varied in different rainfall years. In the present study, mulch with plastic film can inhibit soil evaporation, improve the soil moisture storage, prolong the period of moisture availability, regulating the soil temperature, and promote maize growth, thereby significantly increasing the crop yield and WUE, particular FLSM and FCM treatments. In the long term, the FLSM treatment require high inputs of money and labor every year, but treatments with FCM (furrow planting of maize, separated by consecutive 50 cm wide and 15 cm tall plastic film-mulched ridges) will bring a significant increase income of farmers. Therefore, this treatment can be an innovative practice in maize production in the rainfed area of the Loess Plateau, China, and also applied to other similar semi-arid dryland agricultural regions of the world.

## Author Contributions

The manuscript was reviewed and approved for publication by all authors. XR, ZJ, and QH conceived and designed the experiments. PZ, TW, TC, SA, XR, and QH performed the experiments. PZ, TW, TC, and ZJ analyzed the data. PZ, SA, and XR wrote the paper. PZ, TW, TC, SA, QH, XR, and ZJ reviewed and revised the paper. SA, TW, and ZJ corrected the English language for the paper.

## Conflict of Interest Statement

The authors declare that the research was conducted in the absence of any commercial or financial relationships that could be construed as a potential conflict of interest.
